# Impact of very preterm birth and post-discharge growth on cardiometabolic outcomes at school age: a retrospective cohort study

**DOI:** 10.1186/s12887-021-02851-5

**Published:** 2021-08-31

**Authors:** Jungha Yun, Young Hwa Jung, Seung Han Shin, In Gyu Song, Young Ah Lee, Choong Ho Shin, Ee-Kyung Kim, Han-Suk Kim

**Affiliations:** 1 Department of Pediatrics, Seoul National University Children’s Hospital, Seoul National University College of Medicine, 101, Daehak-ro, Jongno-gu, Seoul, Republic of Korea; 2 Present address: Department of Pediatrics, CHA Ilsan Medical Center, Goyang-si, Republic of Korea; 3 Present address: Department of Pediatrics, Seoul National University Bundang Hospital, Sungnam-si, Republic of Korea; 4 Present address: Department of Pediatrics, Korea University Guro Hospital, Seoul, Republic of Korea

**Keywords:** Very preterm infants, Insulin resistance, Hypertension, Growth

## Abstract

**Background:**

Adverse metabolic outcomes later in life have been reported among children or young adults who were born as preterm infants. This study was conducted to examine the impact of very preterm/very low birth weight (VP/VLBW) birth and subsequent growth after hospital discharge on cardiometabolic outcomes such as insulin resistance, fasting glucose, and systolic and diastolic blood pressure (BP) among children at 6–8 years of age.

**Methods:**

This retrospective cohort study included children aged 6–8 years and compared those who were born at < 32 weeks of gestation or weighing < 1,500 g at birth (*n* = 60) with those born at term (*n* = 110). Body size, fat mass, BP, glucose, insulin, leptin, adiponectin, and lipid profiles were measured. Weight-for-age z-score changes between discharge and early school-age period were also calculated, and factors associated with BP, fasting glucose, and insulin resistance were analyzed.

**Results:**

Children who were born VP/VLBW had significantly lower fat masses, higher systolic BP and diastolic BP, and significantly higher values of fasting glucose, insulin, and homeostatic model assessment of insulin resistance (HOMA-IR), compared to children born at term. VP/VLBW was correlated with HOMA-IR and BPs after adjusting for various factors, including fat mass index and weight-for-age z-score changes. Weight-for-age z-score changes were associated with HOMA-IR, but not with BPs.

**Conclusions:**

Although children aged 6–8 years who were born VP/VLBW showed significantly lower weight and fat mass, they had significantly higher BPs, fasting glucose, HOMA-IR, and leptin levels. The associations of VP/VLBW with cardiometabolic factors were independent of fat mass and weight gain velocity.

**Supplementary Information:**

The online version contains supplementary material available at 10.1186/s12887-021-02851-5.

## Background

Prematurity accounts for almost 10 % of births worldwide and 7.2 % of births in Korea with increasing tendency [1, 2]. Despite improvements in the overall survival of preterm infants in recent decades [3, 4], health inequalities emerge later in life in this population. Since the term “thrift phenomenon” was coined by Barker and colleagues [5], an association between low birth weight or small for gestational age (SGA) babies and cardiometabolic problems, such as insulin resistance and increased blood pressure (BP), has been consistently demonstrated by many prospective and cohort studies [6–8]. Insulin resistance plays an important role in the development of metabolic syndrome [9].

For preterm births, the association between cardiovascular diseases and metabolic syndrome in later life has been demonstrated in several studies [10–13]. In very preterm infants or extremely preterm infants, these cardiometabolic changes typically begin during infancy or school age [14–16]. A cohort study from New Zealand reported that extreme prematurity and high body mass index (BMI) at age 7–8 years were significant predictors of metabolic syndrome in young adulthood [6]. Obesity and accelerated postnatal growth during childhood were also suggested as risk factors for adverse cardiometabolic outcomes in children born with very low birthweight [17]. However, it is not typical for children who were born prematurely to be fat or obese during childhood, even though they have cardiometabolic risks during this period [18, 19]. So far, the association between preterm birth and cardiometabolic risks at early school age, considering adiposity and growth velocity, has been scarcely studied.

This study aimed to investigate whether there are differences in the cardiometabolic factors such as insulin resistance, fasting glucose, and systolic and diastolic BPs in early school-aged children born prematurely compared to term infants and whether these adverse cardiometabolic findings are associated with adiposity and growth velocity.

## Methods

### Study design

This was a retrospective cohort study evaluating children at 6–8 years of age born as very preterm (VP) or very low birth weight (VLBW) and term infants. Preterm infants with a gestational age (GA) < 32 weeks (VP) or birth weight < 1,500 g (VLBW) that were born between 2008 and 2009 in Seoul National University Hospital (*n* = 266) were screened and the families of 136 infants were contacted, where contact information was available. Sixty of these consented for their children to participate in the study. Children with congenital anomalies and chromosomal abnormalities were excluded from the study. There was an existing healthy children cohort operated from the hospital (cohort IRB No. H 1102-097-357) and participants in the cohort were enrolled into the term control group, if they were born at term and were at the age of 6–8 years during the study period. This study was approved by the Institution of Review Board of Seoul National University Hospital (IRB No. H1509-030-702). The study protocol was in accordance with the Declaration of Helsinki.

### Data collection and laboratory analysis

Data on perinatal factors, including birth weight, gestational age (GA), delivery mode, and sex, were reviewed from electronic medical records. SGA was defined as a birth weight < 10th percentile for age according to the latest Fenton growth charts [20]. At 6–8 years of age, body measurements, including weight, height, and waist circumference, were taken, and BMI was calculated based on WHO Child Growth standards [21]. Fat mass and fat free mass were measured using the InBody Test (BIA; InBody 770, Biospace Co., Seoul, Korea). BP was measured around the left upper arm using an appropriately sized cuff and an automated blood pressure measuring device, and the average of two blood pressure readings was calculated. Questionnaires, completed by the parents, were used to assess recent daily intakes of total energy and physical activity. The self-reported BMI of the parents was also collected at the time of evaluation.

Blood was obtained by venipuncture after fasting for at least 8 h. Fasting glucose, insulin, lipid profile, leptin (Human Leptin RIA kit, LINCO Research, Inc., St. Charles., U.S.A.), and adiponectin (Human Adiponectin ELISA, Biovendor, Czech Republic) were measured. Insulin resistance was assessed using the Homeostasis Model Assessment of Insulin Resistance (HOMA-IR), which was calculated as the product of the fasting plasma insulin level multiplied by the fasting plasma glucose level [HOMA-IR = (fasting plasma insulin [U/mL] × fasting plasma glucose [mg/dL])/(22.5 × 18.182)] [22].

To measure growth velocity in the study population, the change in weight-for-age z-score between discharge at term equivalent age from the neonatal intensive care unit and at 6–8 years of age was taken as a variable for VP/VLBW infants. For the term group, change in weight-for-age z-score between birth and early school age was calculated because preterm infants experience postnatal growth restriction during their stay in the neonatal intensive care unit. The study population in each group was further categorized according to the growth velocity from birth in term infants or from discharge at term equivalent age in preterm infants to 6–8 years of age, such as those with an increase in the weight-for-age z-scores (z-score change > 0) and those with a decrease in the weight-for-age z-scores (z-score change ≤ 0), for the subgroup analysis of HOMA-IR, fasting glucose, and systolic and diastolic BP at school age.

### Statistical analysis

Data analysis was performed using STATA 12.0 for Windows (Stata Corp, College Station, TX, USA). The Wilcoxon rank sum test was used for the comparison of continuous variables, and Fisher’s exact test was used for categorical variables. HOMA-IR and leptin were skewed and transformed onto a logarithmic scale before analysis. Correlations between variables and cardiometabolic outcomes such as fasting glucose, HOMR-IR, and systolic and diastolic BP at school age were evaluated using Pearson’s correlation coefficient and regression analyses. Three models of partial correlation analysis were conducted to measure the effect of VP/VLBW birth on cardiometabolic outcomes. In Model I, VP/VLBW birth, age, sex, SGA, mode of delivery, and any breast milk feeding > 6 months were included in the multivariate regression analysis for cardiometabolic outcomes. In Model II, lean mass index (LMI) and fat mass index (FMI) were additionally included in the multivariate analysis, and in a third model, the weight-for-age z-score change was additionally adjusted to explore the effect of the weight-for-age z-score change on the cardiometabolic outcomes (Model III). Listwise deletion was used to handle missing data during multiple regression analysis, which resulted in excluding the data of 31 infants without information on breastfeeding, one infant without measurement of fat mass, and one infant without measurement of the insulin level. Sensitivity analysis was conducted excluding SGA infants among the term and VP/VLBW infants; the results are provided in the Supplementary Materials. *P*-values < 0.05 were considered statistically significant. Data are presented as the median (interquartile range) or rates.

## Results

### Demographic findings of the study population

Sixty VP/VLBW and 110 term infants were included in the study. Median GA and birth weight were 28.4 weeks (interquartile range, 26.3–30.5 weeks) and 935 g (790-1,220 g) in the VP/VLBW group and 39.6 weeks (38.4–40.3 weeks) and 3,240 g (2,970-3,500 g) in the term group (Table 1). Median postmenstrual age and weight at discharge were 37.9 weeks (36.1–40.6 weeks) and 2,300 g (1,960-2,580 g) in the VP/VLBW infants. There were significantly more SGA infants and Cesarean section deliveries in the VP/VLBW group. The incidence of breastmilk feeding at > 6 months of age was significantly greater in the term group. There were no significant differences in parental education level and parental BMI between the two groups.

**Table 1:** Demographic and parental information of the study population

	VP/VLBW (*n* = 60)	Term (*n* = 110)	*p*-value
GA at birth (weeks)	28.4 (26.3–30.5)	39.6 (38.4–40.3)	< 0.001
Birth weight (g)	935 (790–1220)	3240 (2970–3500)	< 0.001
Birth weight z-score	-0.2 (-1.3-0.3)	-0.2 (-0.9-0.2)	0.388
SGA	15 (25.0)	9 (8.2)	0.005
Female	28 (46.7)	57 (51.8)	0.630
Cesarean section	41 (68.3)	35 (31.8)	< 0.001
PMA at discharge (weeks)	37.9 (36.1–40.6)	-	-
Weight at discharge (g)	2300 (1960–2580)	-	-
Weight-for-age z-score at discharge	-1.7 (-3.3–1.0)	-	-
Any breastmilk feeding > 6 months	24 (46.2)	67 (77)	< 0.001
Paternal education college or above	48 (80.0)	87 (79.1)	1.000
Maternal education college or above	48 (80.0)	90 (81.8)	0.838
Paternal BMI (kg/m^2^)	24.8 (22.1–25.9)	24.4 (22.8–26.4)	0.573
Maternal BMI (kg/m^2^)	21.1 (19.1–22.6)	21.5 (19.7–24)	0.146

Values are expressed as n (%) or median (interquartile range). The Wilcoxon rank sum test was used for the comparison of continuous variables, and Fisher’s exact test was used for categorical variables

*VP/VLBW* very preterm/very low birthweight, *GA* gestational age, *SGA* small for gestational age, *BMI* body mass index, *PMA* postmenstrual age

### Body measurements at early school age

The median age at evaluation was 7.2 years (6.8–7.5 years) in the VP/VLBW group and 7.8 years (6-7.9 years) in the term group (*p* = 0.801) (Table 2). The z-scores for weight-for-age and height-for-age were significantly lower in the VP/VLBW group. Although the BMI was significantly lower in the VP/VLBW group (15.0 vs. 15.5 kg/m^2^, *p* = 0.003), the BMI-for-age z-scores were comparable between the two groups (-0.5 vs. -0.3, *p *= 0.07). Lean body mass (18.4 vs. 19.2 kg,* p* = 0.18) and lean mass index (12.6 vs. 12.9 kg/m^2^, *p* = 0.09) were also comparable between the two groups. Fat mass (3.1 vs. 4.1 kg, *p* = 0.002) and fat mass index (2.1 vs. 2.8 kg/m^2^, *p* = 0.002) were significantly lower in the VP/VLBW group. Daily caloric intake, weekly physical activity, and walk hours were comparable between the two groups.

**Table 2:** Body measurements in early school-aged children

	VP/VLBW (*n* = 60)	Term (*n* = 110)	*p*-value
Age (years)	7.2 (6.8–7.5)	7.8 (6.0-7.9)	0.801
Weight (kg)	20.8 (19.5–24.8)	23.0 (19.9–26.9)	0.028
Weight-for-age z-score	-0.8 (-1.3-0.1)	0.0 (-0.8-0.9)	0.001
Change in weight-for-age z-score	1.4 (0.2–2.8)	0.2 (-0.4-0.9)	< 0.001
Height-for-age z-score	-0.3 (-1.0-0.1)	0.2 (-0.2-0.9)	< 0.001
BMI (kg/m^2^)	15.0 (13.9–15.9)	15.5 (14.6–16.9)	0.003
BMI-for-age z-score	-0.5 (-1.5-0.2)	-0.3 (-1-0.5)	0.072
Lean body mass (kg)	18.4 (16.9–20.2)	19.2 (17.1–21.2)	0.180
Lean mass index (kg/m^2^)	12.6 (12.0-13.1)	12.9 (12.3–13.4)	0.091
Fat mass (kg)	3.1 (2.1–4.1)	4.1 (2.8–5.8)	0.002
Fat mass index (kg/m^2^)	2.1 (1.6–2.9)	2.8 (1.9–3.9)	0.002
Waist circumference (cm)	51.6 (49.0-54.3)	53.8 (50.0–57.0)	0.013
Calorie intake (kcal/day)	1537 (1345.9-1709.4)	1441 (1299.4-1588.1)	0.114
Moderate or more activity^a^	120 (20–320)	180 (70–300)	0.140
Walking time^a^	120 (30–210)	120 (60–210)	0.403

Values are expressed as the median (interquartile range)

*VP/VLBW* very preterm/very low birthweight, *BMI* body mass index, *BP* blood pressure

^a^Values are expressed as minutes/week. The Wilcoxon rank sum test was used

### Blood pressures and laboratory findings

Systolic and diastolic BPs were significantly higher in the VP/VLBW group (107 vs. 97.5 mmHg, *p* < 0.001, and 64 vs. 59 mmHg, *p* < 001, respectively) (Table 3). In comparison to children born at term, children born as VP/VLBW had significantly higher high-density lipoprotein (HDL) cholesterol, fasting glucose, and HOMA-IR. Leptin was also significantly higher in the VP/VLBW group, while adiponectin was comparable between the two groups.

**Table 3:** Blood pressures and laboratory findings of metabolic syndrome

	VP/VLBW (*n* = 60)	Term (*n* = 110)	*p*-value
Systolic BP (mmHg)	107 (102.5–111.0)	97.5 (93.0-102.0)	< 0.001
Diastolic BP (mmHg)	64 (59.3–68.5)	59 (54.5–64.0)	< 0.001
HDL-Cholesterol (mg/dL)	69 (61–79)	64 (55–73)	0.010
TG (mg/dL)	60 (46–74)	57.5 (48–74)	0.844
Fasting glucose (mg/dL)	96 (93–101)	93 (88–97)	< 0.001
Fasting insulin (µIU/mL)	4.4 (3.4–5.9)	3.7 (2.6–5.8)	0.053
HOMA-IR	1.1 (0.81–1.49)	0.8 (0.58–1.35)	0.017
Leptin (ng/mL)	6.4 (5.1–10.0)	5.8 (4.0-9.3)	0.040
Adiponectin (µg/mL)	9.7 (7.2–11.5)	8.9 (6.6–10.9)	0.297

Values are expressed as the median (interquartile range). The Wilcoxon rank sum test was used for the comparison of continuous variables, and Fisher’s exact test was used for categorical variables

*VP/VLBW* very preterm/very low birthweight, *BP* blood pressure. *HDL-Cholesterol* high-density lipoprotein cholesterol, *TG* triglyceride, *HOMA-IR* homeostasis model assessment of insulin resistance

### Cardiometabolic outcomes among VP/VLBW and term infants according to growth velocity

**Fig. 1:**
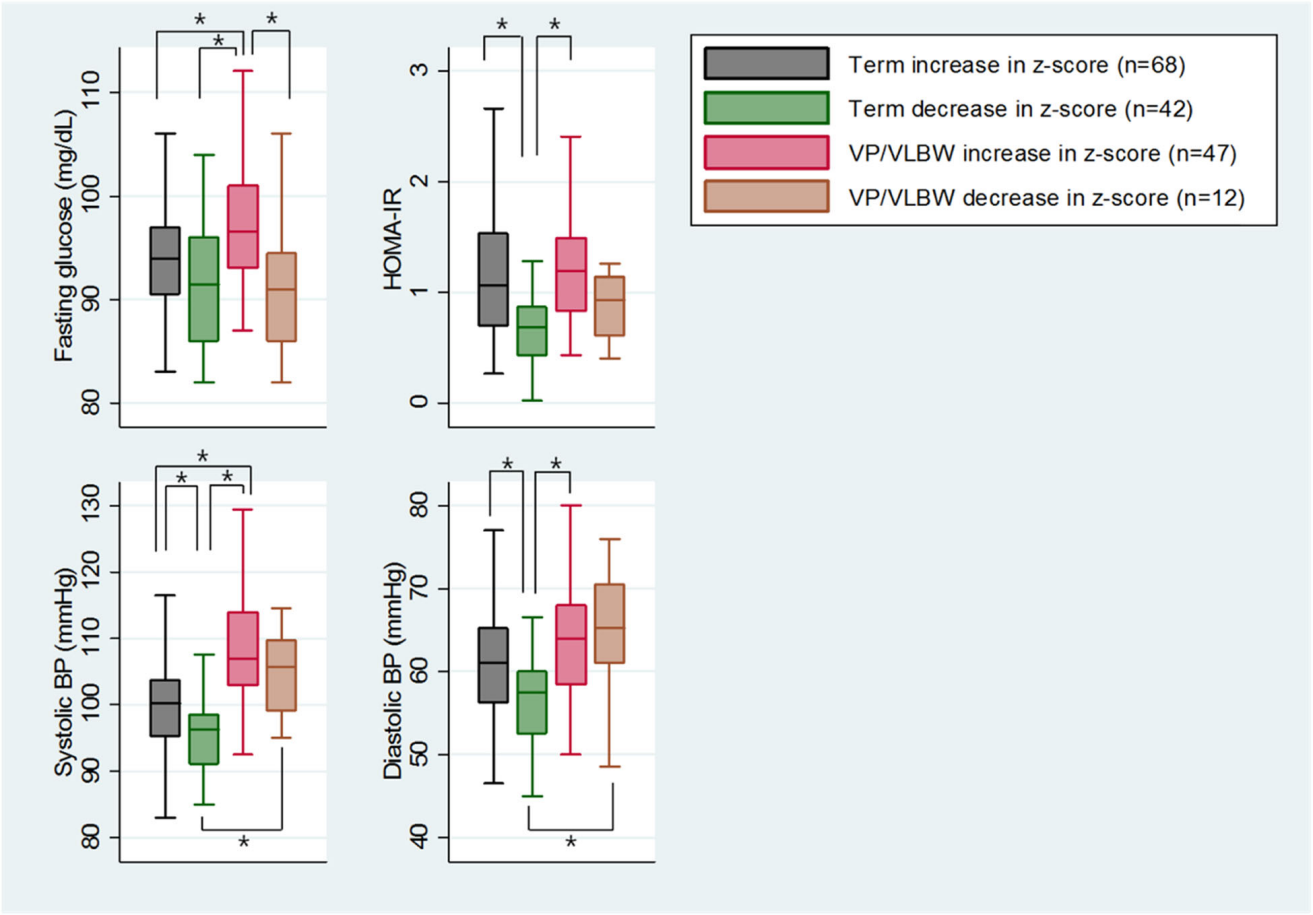
Cardiometabolic outcomes of term and VP/VLBW infants according to growth pattern. VP/VLBW infants with an increase in weight-for-age z-scores showed significantly higher fasting glucose, HOMA-IR, and systolic and diastolic BPs compared to term infants with a decrease in weight-for-age z-scores and also showed significantly higher fasting glucose and systolic BP compared to term infants with an increase in weight-for-age z-scores. VP/VLBW infants with a decrease in weight-for-age z-scores had a significantly higher systolic and diastolic BP than term infants with a decrease in weight-for-age z-scores but showed no significant differences in HOMA-IR and fasting glucose compared to term infants regardless of z-score changes. *VP/VLBW* very preterm/very low birthweight, *HOMA-IR* homeostasis model assessment of insulin resistance, *BP* blood pressure. ANOVA with a Bonferroni test was conducted and *represents a significant difference between groups (*P* < 0.05)

When VP/VLBW and term infants were further categorized according to the growth pattern, VP/VLBW infants with an increase in weight-for-age z-scores had significantly higher HOMA-IR, fasting glucose, and systolic and diastolic BPs than term infants with a decrease in weight-for-age z-scores and had significantly higher fasting glucose and systolic BP than term infants with an increase in weight-for-age z-scores (Fig. 1). However, VP/VLBW infants with a decrease in weight-for-age z-scores showed no significant differences in HOMA-IR and fasting glucose compared to term infants regardless of z-score changes, while systolic and diastolic BPs in VP/VLBW infants with a decrease in z-scores were significantly higher than in term infants with a decrease in weight-for-age z-scores.

Among the VP/VLBW infants, fasting glucose was significantly higher in those with an increase in weight-for-age z-scores than in those with a decrease in weight-for-age z-scores. In term infants, HOMA-IR and systolic and diastolic BP were significantly higher in those with an increase in weight-for-age z-scores than in those with a decrease in z-scores.

### Association of preterm birth and weight-for-age z-score change with cardiometabolic outcomes

In a simple regression analysis, VP/VLBW was associated with higher systolic and diastolic BP, fasting glucose, and HOMA-IR (Table 4). Partial correlation analysis without adjusting for LMI, FMI, and weight-for-age z-score change (Model I) showed that VP/VLBW was associated with higher fasting glucose, HOMA-IR, and systolic and diastolic BP (Table S1). In Model II, which additionally adjusted for LMI and FMI, all four outcomes were associated with VP/VLBW and FMI was associated with HOMA-IR and diastolic BP (Table S2). In Model III, which additionally adjusted for change in the weight-for-age z-score as well as LMI and FMI, VP/VLBW was associated with HOMA-IR, systolic and diastolic BP (Table 4).

**Table 4:** Correlation analysis for fasting glucose, HOMA-IR, and systolic and diastolic BP (Model III)

	Fasting glucose				HOMA-IR^a^				Systolic BP				Diastolic BP			
	Univariate		Multivariate		Univariate		Multivariate		Univariate		Multivariate		Univariate		Multivariate	
	coef	SE	coef	SE	coef	SE	coef	SE	coef	SE	coef	SE	coef	SE	coef	SE
VP/VLBW	3.82^§^	0.98	1.88	1.39	0.28^§^	0.11	0.28^¶^	0.13	9.17^§^	1.22	8.28^§^	1.73	4.46^§^	1.32	6.05^§^	1.85
Age (years)	1.35^¶^	0.60	0.93	0.65	0.20^§^	0.06	0.17^§^	0.06	2.02^¶^	0.83	1.27	0.82	2.91^§^	0.78	2.19^¶^	0.88
Female	-3.52^§^	0.94	-2.56^¶^	1.14	0.10	0.10	-0.03	0.11	1.48	1.34	2.04	1.43	0.43	1.30	0.29	1.53
LMI (kg/m^2^)	1.02	0.56	0.15	0.85	0.17^§^	0.06	-0.14	0.08	0.13	0.79	-0.73	1.06	1.17	0.76	-0.08	1.14
FMI (kg/m^2^)	0.12	0.23	-0.46	0.34	0.13^§^	0.02	0.07^¶^	0.03	0.55	0.31	0.59	0.43	1.11^§^	0.29	1.66^§^	0.46
Weight-for-age z-score change	1.62^§^	0.34	1.77^§^	0.54	0.22^§^	0.04	0.19^§^	0.05	2.24^§^	0.47	0.61	0.67	1.08^¶^	0.48	-0.83	0.72

Adjusted for preterm birth, age, sex, small for gestational age, mode of delivery, lean mass index, fat mass index, any breastmilk feeding >6 months and weight-for-age z-score change

*Coef* correlation coefficient, *SE* standard error, *HOMA-IR* homeostatic model assessment-insulin resistance, *BP* blood pressure, *VP/VLBW* very preterm/very low birthweight, *LMI* lean mass index, *BMI* body mass index

^a^Transformed onto a logarithmic scale

^¶^and ^§^ represents significant correlations (*p* < 0.05 and *p* < 0.01, respectively)

Weight-for-age z-score change was associated with fasting glucose, HOMA-IR, and systolic and diastolic BP in the simple regression analysis (Table 4). Multivariate analysis showed that systolic and diastolic BP were no longer associated with the weight-for-age z-score change, while fasting glucose and HOMA-IR remained associated with the weight-for-age z-score change (Model III).

Regression analysis was also conducted after excluding the SGA infants from the study population. The results showed that VP/VLBW was also associated with systolic and diastolic BP in both the simple regression and partial correlation analysis (Table S4). Fasting glucose and HOMA-IR were not associated with VP/VLBW in the multivariate analysis, while weight-for-age z-score change was significantly associated with fasting glucose and HOMA-IR.

## Discussion

In this retrospective cohort study, VP/VLBW infants were compared with term infants for cardiometabolic outcomes at school age, and important factors associated with outcomes such as post-discharge growth, weight, lean body mass, and fat mass were considered and adjusted. Early school-aged children who were born as VP/VLBW had higher BP, more insulin resistance, and higher fasting glucose than those who were term infants, while weight and fat mass were lower in the VP/VLBW group. Insulin resistance and BPs were consistently associated with VP/VLBW, independent of LMI, FMI, and weight-for-age z-score change, as shown in all three models in the multivariate analysis. The weight-for-age z-score change from the neonatal period to school age was not associated with BPs but did have a significant correlation with insulin resistance and fasting glucose in the multivariate analysis.

Patterns of growth, as well as alterations in adipose tissue, were different between SGA infants and preterm infants. Previous SGA studies have reported that these infants had less adipose tissue at birth, becoming similar after the neonatal period [23], and a higher fat distribution was observed in childhood and adolescence [24, 25]. However, for the preterm infants, lower BMI and fat mass were frequently seen in infancy and school age compared with term infants [26, 27], even though they had higher BMIs and fat mass with adverse cardiometabolic outcomes in adulthood [28–31].

Although the interaction between obesity and insulin resistance is a key pathogenesis factor in the development of metabolic syndrome [32], cardiometabolic problems in preterm infants at school age have been reported, despite a lower BMI during this period [15, 33]. In the present study, to elucidate the effect of body composition more clearly, fat mass and lean body mass were measured and adjusted, and the results showed that preterm birth was associated with increased BP, fasting glucose, and HOMA-IR, independent of LMI and FMI (Models I and II).

Moreover, as catch-up growth is an important factor in the development of insulin resistance [34], weight-for-age z-score change until school age was considered and adjusted, and it was found to be associated with insulin resistance. The weight-for-age z-score was much lower in preterm infants at discharge compared to term infants, with a subsequent increase by early school age, resulting in a higher weight gain velocity in preterm infants in this study, even though the weight-for-age z-score was still lower than that of term infants at 6–8 years of age. As with SGA infants [35, 36], preterm infants experienced the development of insulin resistance during rapid weight gain [37].

To explore the role of weight gain velocity in the development of a cardiometabolic problem, a third multivariate analysis was conducted. In Model III, the weight-for-age z-score change was additionally adjusted for and the results showed that preterm birth was associated with HOMA-IR, and systolic and diastolic BP. Fasting glucose was no longer associated with preterm birth and the coefficient of preterm birth for HOMA-IR became smaller than in the first two models, while the coefficient of preterm birth for systolic and diastolic BP was similar or higher compared with the other two models. These interactions of the weight-for-age z-score change with other confounding factors and cardiometabolic outcomes suggested that glucose and insulin metabolism could be mediated by weight gain velocity in preterm infants.

In contrast, while higher BPs were associated with preterm birth in the three multivariate models, the weight-for-age z-score change was not associated with BPs at school age in the multivariate analysis. Although the mechanisms of elevated BP in children born prematurely are not fully understood, impaired development of the glomeruli with decreased nephrons, microvascular growth arrest, and sympathoadrenal overactivity might be contributing factors [14, 38, 39]. These conditions were associated with preterm birth and related morbidities during a neonatal intensive care unit stay, rather than the pattern of growth beyond the neonatal period. However, the influence of weight gain velocity on increased BP should not be ignored because a longitudinal cohort study from the UK showed that growth gain velocity from 1 year of age until adolescence was correlated with systolic and diastolic BP [40].

When preterm infants were further categorized according to their growth velocity, cardiometabolic factors were consistently higher in the preterm infants with improved growth group, compared to term infants with negative growth. However, there were no differences in the HOMA-IR and fasting glucose levels between the term infants with or without positive growth and the preterm infants with negative growth, and fasting glucose was lower in the preterm infant group with negative growth than in the group with positive growth. Interestingly, systolic and diastolic BPs were higher in both preterm infant groups, regardless of growth velocity after discharge, than in the term infants with negative growth and there were no differences in the systolic and diastolic BPs between the two preterm groups. Notwithstanding the small sample sizes used for these subgroup analyses, they did demonstrate an impact of the growth pattern in preterm infants on adverse cardiometabolic findings, as shown in the multivariate analysis.

There are several limitations to our study. A relatively small patient population was analyzed, and the growth velocity of the early post-discharge period, such as the time between discharge and 1 year of age, was not compared. Also, the fat mass was not measured by dual energy X-ray absorptiometry (DEXA). However, bioelectrical impedance analysis is a useful method for estimating body composition and has been used in both clinical and research fields in the pediatric population [41, 42]. The aforementioned New Zealand Very Low Birth Weight Study also used bioelectrical impedance as a method to measure fat mass [6].

## Conclusions

In this study, VP/VLBW was found to represent an important risk factor for elevated BP at 6–8 years, independent of weight, fat mass, and growth velocity after discharge. VP/VLBW, as well as growth velocity, was also related to increased insulin resistance at 6–8 years. Despite the low weight and low fat mass, children born prematurely may be at a higher risk for increased BP, and VP/VLBW infants with greater weight gain after discharge may be at risk of insulin resistance during early school age. Further studies are required to investigate the growth pattern of VP/VLBW infants, including detailed fat distribution, to determine an appropriate growth in this population to minimize the risk of adverse cardiometabolic outcomes.

## Supplementary Information

**Additional file 1: Table S1.** Multivariate analysis for cardiometabolic findings without adjusting weight-for-age z-score change, LMI and FMI (Model I). **Table S2.** Multivariate analysis for cardiometabolic findings without adjusting weight-for-age z-score change (Model II). **Table S3.** Body measurements in early school-aged children who were born as appropriate for gestational age. **Table S4.** Blood pressures and laboratory findings of metabolic syndrome among school-aged children who were born as appropriate for gestational age. **Table S5. **Partial correlation analysis for HOMA-IR and systolic and diastolic BP school-aged children who were born as appropriate for gestational age

## Data Availability

The datasets generated and analyzed are not publicly available but are available from the corresponding author on reasonable request.

## Abbreviations

BMI: Body mass index
BP: Blood pressure
HOMA-IR: Homeostatic Model Assessment of Insulin Resistance
SGA: Small for gestational age
GA: Gestational age
PMA: Postmenstrual age
HDL: High-density lipoprotein
DEXA: Dual energy X-ray absorptiometry
TG: Triglyceride
