# Supplementary material for: Impact of very preterm birth and post-discharge growth on cardiometabolic outcomes at school age: a retrospective cohort study
Source: BMC Pediatr. 2021 Aug 31;21:373. doi: 10.1186/s12887-021-02851-5 (PMC8406828; doi:10.1186/s12887-021-02851-5)
Supplement: Supplementary file 1 — Additional file 1: Table S1. Multivariate analysis for cardiometabolic findings without adjusting weight-for-age z-score change, LMI and FMI (Model I). Table S2. Multivariate analysis for cardiometabolic findings without adjusting weight-for-age z-score change (Model II). Table S3. Body measurements in early school-aged children who were born as appropriate for gestational age. Table S4. Blood pressures and laboratory findings of metabolic syndrome among school-aged children who were born as appropriate for gestational age. Table S5. Partial correlation analysis for HOMA-IR and systolic and diastolic BP school-aged children who were born as appropriate for gestational age [file 12887_2021_2851_MOESM1_ESM.docx]

**Impact of very preterm birth and post-discharge growth on cardiometabolic outcomes at school age: a retrospective cohort study**

Jungha Yun^1#,a^, Young Hwa Jung^1#,b^, Seung Han Shin^1*^, In Gyu Song^1,c^, Young Ah Lee^1^, Choong Ho Shin^1^, Ee-Kyung Kim^1^, Han-Suk Kim^1^

^1^Department of Pediatrics, Seoul National University Children’s Hospital, Seoul National University College of Medicine, Seoul, Republic of Korea

^#^Jungha Yun and Young Hwa Jung contributed equally to this work.

Current address:

^a^Department of Pediatrics, CHA Ilsan Medical Center, Goyang-si, Republic of Korea

^b^Department of Pediatrics, Seoul National University Bundang Hospital, Sungnam-si, Republic of Korea

^c^Department of Pediatrics, Korea University Guro Hospital, Korea University College of Medicine, Seoul, Republic of Korea

**^*^Corresponding Author:** Seung Han Shin, Ph.D., Department of Pediatrics, Seoul National University College of Medicine, 101, Daehak-ro, Jongno-gu, Seoul, Republic of Korea, Tel.: +82 2 2072 3555, Fax: +82 2 2072 0590, E-mail: [revival421@snu.ac.kr](mailto:revival421@snu.ac.kr)

Table S1. Multivariate analysis for cardiometabolic findings without adjusting weight-for-age z-score change, LMI and FMI (Model I)

|  | Fasting glucose | | HOMA-IR* | | Systolic BP | | Diastolic BP | |
| --- | --- | --- | --- | --- | --- | --- | --- | --- |
|  | coef | SE | coef | SE | coef | SE | coef | SE |
| VP/VLBW | 4.13^§^ | 1.25 | 0.42^§^ | 0.13 | 8.42^§^ | 1.50 | 4.20^¶^ | 1.67 |
| Age (year) | 1.15 | 0.67 | 0.20^§^ | 0.07 | 1.40 | 0.81 | 2.46^§^ | 0.90 |
| Female | -3.06^§^ | 1.06 | 0.01 | 0.11 | 2.41 | 1.26 | 0.70 | 1.41 |

Adjusted for preterm birth, age, sex, small for gestational age, mode of delivery, and any breastmilk feeding >6 months. * Transformed onto a logarithmic scale. HOMA-IR, Homeostatic model assessment-insulin resistance; BP, blood pressure; VP/VLBW, very preterm/very low birthweight. ^¶^and ^§^ represents a significant correlations (p<0.05 and p<0.01, respectively).

Table S2. Multivariate analysis for cardiometabolic findings without adjusting weight-for-age z-score change (Model II)

|  | Fasting glucose | | HOMA-IR* | | Systolic BP | | Diastolic BP | |
| --- | --- | --- | --- | --- | --- | --- | --- | --- |
|  | coef | SE | coef | SE | coef | SE | coef | SE |
| VP/VLBW | 4.23^§^ | 1.26 | 0.51^§^ | 0.12 | 8.94^§^ | 1.51 | 5.24^§^ | 1.62 |
| Age (year) | 1.00 | 0.68 | 0.18^§^ | 0.07 | 1.28 | 0.82 | 2.18^¶^ | 0.87 |
| Female | -2.89^¶^ | 1.17 | -0.05 | 0.11 | 2.03 | 1.40 | 0.24 | 1.51 |
| LMI (kg/m^2^) | 0.66 | 0.84 | -0.07 | 0.08 | -0.46 | 1.01 | -0.51 | 1.09 |
| FMI (kg/m^2^) | 0.05 | 0.32 | 0.13^§^ | 0.03 | 0.76 | 0.39 | 1.44^§^ | 0.42 |

Adjusted for preterm birth, age, sex, small for gestational age, mode of delivery, lean mass index, fat mass index, and any breastmilk feeding >6 months. * Transformed onto a logarithmic scale. HOMA-IR, Homeostatic model assessment-insulin resistance; BP, blood pressure; VP/VLBW, very preterm/very low birthweight; LMI, lean mass index; BMI, body mass index. ^¶^and ^§^ represents a significant correlations (p<0.05 and p<0.01, respectively).

| Table S3. Body measurements in early school-aged children who were born as appropriate for gestational age | | | |
| --- | --- | --- | --- |
|  | VP/VLBW (n=45) | Term (n=101) | p value |
| Age (year) | 7.2 (6.8-7.5) | 7.8 (6-7.9) | 0.691 |
| Weight (kg) | 21.2 (20-24.9) | 23.5 (20-27.1) | 0.137 |
| Weight-for-age z-score | -0.6 (-1.1-0.2) | 0 (-0.7-0.9) | 0.017 |
| Change in weight-for-age z-score | 1.3 (0.1-2) | 0.2 (-0.4-0.8) | <0.001 |
| Height-for-age z-score | -0.1 (-0.6-0.4) | 0.2 (-0.2-0.9) | <0.001 |
| BMI (kg/m^2^) | 15 (14-16) | 15.7 (14.8-16.9) | 0.032 |
| BMI-for-age z-score | -0.3 (-1-0.4) | -0.2 (-0.8-0.5) | 0.320 |
| Lean body mass (kg) | 18.7 (17.3-20.8) | 19.3 (17.2-21.5) | 0.504 |
| Lean mass index (kg/m^2^) | 12.7 (12.2-13.3) | 12.9 (12.3-13.4) | 0.310 |
| Fat mass (kg) | 3.1 (2.6-4.1) | 4.2 (3.1-5.8) | 0.006 |
| Fat mass index (kg/m^2^) | 2.2 (1.7-2.9) | 2.8 (2-4) | 0.008 |
| Waist circumference (cm) | 51.8 (50-54.5) | 54 (50.6-57) | 0.038 |
| Calorie intake (Kcal/day) | 1538 (1367.9-1694.9) | 1440 (1299.4-1578.4) | 0.070 |
| Moderate or more activity ^a^ | 120 (20-250) | 180 (70-300) | 0.080 |
| Walking time ^a^ | 90 (30-210) | 120 (60-210) | 0.383 |

Values are expressed as the median (interquartile range). The Wilcoxon rank sum test was used for the comparison of continuous variables, and Fisher’s exact test was used for categorical variables.

*BMI* body mass index

^a^values expressed as minutes/week

Table S4. Blood pressures and laboratory findings of metabolic syndrome among school-aged children who were born as appropriate for gestational age

|  | VP/VLBW (n=45) | Term (n=101) | p value |
| --- | --- | --- | --- |
| Systolic BP (mmHg) | 107 (103-110.5) | 97.5 (92.5-103) | <0.001 |
| Diastolic BP (mmHg) | 64 (59.5-68.5) | 59 (55-64) | <0.001 |
| HDL-Cholesterol (mg/dl) | 70 (61-79) | 64 (55-73) | 0.029 |
| TG (mg/dl) | 60 (45-73) | 57 (48-74) | 0.906 |
| Fasting glucose (mg/dl) | 96 (93-101) | 93 (88-97) | 0.001 |
| Fasting insulin (mU/ml) | 4.5 (3.5-5.9) | 3.8 (2.7-5.8) | 0.081 |
| HOMA-IR | 1.1 (0.81-1.5) | 0.9 (0.62-1.35) | 0.042 |
| Leptin (ng/ml) | 6.4 (5-8.4) | 5.9 (4.2-9.3) | 0.168 |
| Adiponectin (μg/mL) | 9.7 (7.7-11.8) | 8.9 (6.7-10.9) | 0.143 |

Values are expressed as the median (interquartile range). The Wilcoxon rank sum test was used for the comparison of continuous variables, and Fisher’s exact test was used for categorical variables.

*BP* blood pressure. *HDL-Cholesterol* high-density lipoprotein cholesterol, *TG* triglyceride, *HOMA-IR* Homeostasis model assessment of insulin resistance.

Table S5. Partial correlation analysis for HOMA-IR and systolic and diastolic BP school-aged children who were born as appropriate for gestational age

|  | Fasting glucose | | | | HOMA-IR^*^ | | | | Systolic BP | | | | Diastolic BP | | | | | |
| --- | --- | --- | --- | --- | --- | --- | --- | --- | --- | --- | --- | --- | --- | --- | --- | --- | --- | --- |
|  | Univariate | | Multivariate | | Univariate | | Multivariate | | Univariate | | Multivariate | | | Univariate | | Multivariate | | |
|  | coef | SE | coef | SE | coef | SE | coef | SE | Coef | SE | beta | SE | | coef | SE | beta | SE |  |
| VP/VLBW | 3.99^§^ | 1.07 | 2.08 | 1.48 | 0.28^¶^ | 0.12 | 0.22 | 0.15 | 9.44^§^ | 1.36 | 8.29^§^ | 1.87 | | 4.82^§^ | 1.50 | 6.17^§^ | 2.08 |  |
| Age (year) | 1.31^¶^ | 0.63 | 0.99 | 0.69 | 2.22^¶^ | 0.87 | 0.17^¶^ | 0.07 | 2.22^¶^ | 0.87 | 1.48 | 0.87 | | 2.98^§^ | 0.84 | 2.04^¶^ | 0.97 |  |
| Female | -3.60^§^ | 0.99 | -2.48^¶^ | 1.21 | 1.34 | 1.45 | 0.01 | 0.12 | 1.34 | 1.45 | 1.94 | 1.53 | | 0.60 | 1.43 | 0.30 | 1.70 |  |
| LMI (kg/m^2^) | 0.73 | 0.62 | -0.05 | 0.88 | 0.16^¶^ | 0.07 | -0.13 | 0.09 | 0.85 | 0.87 | -0.44 | 1.11 | | 1.24 | 0.86 | -0.03 | 1.23 |  |
| FMI (kg/m^2^) | 0.001 | 0.24 | -0.43 | 0.36 | 0.12^§^ | 0.02 | 0.06 | 0.04 | 0.60^¶^ | 0.33 | 0.46 | 0.45 | | 1.04^§^ | 0.31 | 1.58^§^ | 0.50 |  |
| Weight-for-age z-score change | 1.59^§^ | 0.38 | 1.45^¶^ | 0.59 | 0.26^§^ | 0.04 | 0.20^§^ | 0.06 | 2.49^§^ | 0.54 | 0.66 | 0.75 | | 1.30^¶^ | 0.56 | -0.90 | 0.83 |  |

Adjusted for preterm birth, age, sex, mode of delivery, lean mass index, fat mass index, any breastmilk feeding >6 months and. * Transformed onto a logarithmic scale. Coef, correlation coefficient; SE, standard error; HOMA-IR, Homeostatic mod weight-for-age z-score change el assessment- insulin resistance; BP, blood pressure; VP/VLBW, very preterm/very low birthweight; LMI, lean mass index; BMI, body mass index. ^¶^and ^§^ represents a significant correlations (p<0.05 and p<0.01, respectively).

Questionnaires of neonatal nutrition and physical activity at school-age

1. Basic information

| Name |  | Birthday | | ( / / ) YYYY/MM/DD | | | | | Siblings: birth order (    ) of the total number (    ) | | |
| --- | --- | --- | --- | --- | --- | --- | --- | --- | --- | --- | --- |
| Parents | Paternal Height: (          )cm, Weight (         ) kg  Maternal Height: (          )cm, Weight (         )kg , Gestational DM (□Yes / □No)  Mother's menarche: ( ) years of age | | | | | | | | | | |
| Family history |  | | Father/  Mother | Paternal Grand  father/mother | | | Maternal Grand  father/mother | | Sibling 1/  Sibling 2 | Aunt/  Uncle | |
|  | DM | | □ / □ | □ / □ | | | □ / □ | | □ / □ | □ / □ | |
|  | High cholesterol | | □ / □ | □ / □ | | | □ / □ | | □ / □ | □ / □ | |
|  | Hypertension | | □ / □ | □ / □ | | | □ / □ | | □ / □ | □ / □ | |
|  | Heart disease | | □ / □ | □ / □ | | | □ / □ | | □ / □ | □ / □ | |
|  | Stroke | | □ / □ | □ / □ | | | □ / □ | | □ / □ | □ / □ | |
|  | Unexpected death <55 years | | □ / □ | □ / □ | | | □ / □ | | □ / □ | □ / □ | |
|  | Thyroid disease | | □ / □ | □ / □ | | | □ / □ | | □ / □ | □ / □ | |
| Past history | □ Seizure □ Meningitis □ Brain trauma □ Other chronic disease (                      ) | | | | | | | | | | |
| Medications | □ Nutrients □ Hurbal Medicine □ Vitamins □ Others  (                      ) | | | | | | | | | | |
| Past 2 weeks | Any history of respiratory or gastrointestinal infections?  □Yes / □No | | | | | | | | | | |
| Symptoms | □ Headache | □Vomiting | | | □Nocturia | □Visual disturbance | | □Personality change | | | □Fall down |
| Premature thelarche: □Yes / □No | | | | | | | | | | | |

1. Neonatal and infant nutrition

|  | When did it start | When did it end |
| --- | --- | --- |
| Any breastfeeding?  □Yes / □No | □ (    ) days after birth | □ Colostrum only/ |
|  |  | □ Within 1 month |
|  |  | □ Until (     ) months |
| Breast milk fortifiers?  □Yes / □No | □ (    ) days after birth | □ Until (     ) months |
| Any formula feeding?  □Yes / □No | □ Within 1 month | □ Within 1 month |
|  | □ Since (     ) months | □ Until (     ) months after birth |
| Duration of preterm formula? | □ not applicable | □ Until (     ) months after birth |
| Any dietary supplements for additional calorie?  (other than those mentioned above) | □ not applicable | □ Until (     ) months after birth |
| When did your child have more formula than the breast milk? | □ Within 1 month  □ Since (     ) months | |
| When did your child start weaning? | Since (     ) months | |
| When did your child start to eat like adults? | Since (     ) months | |

3. Does the child have any other diseases? □Yes / □No

If yes, ( )

4. Are there any other medications your child is taking regularly? (recent 2 years)

□Yes ( ) □No

5. Physical activity

1) How many days did your child have more than 10 minutes of vigorous physical activity that requires large amount of oxygen consumption and increases heart rate in the past week?

□ Not at all □ 1 day □ 2 days □ 3 days □ 4 days □ 5 days □ 6 days

□ 7 days (everyday)

| The examples of vigorous physical activity: Jogging (running), soccer, basketball, rope skipping, judo, taekwondo, kendo, rock climbing, mountain climbing, aerobic dance, singles tennis, squash, hockey, roller skating, bicycle (fast), swimming (fast), carrying heavy objects, etc. |
| --- |

How many minutes per day did your child usually do this vigorous physical activity?

( ) hours ( ) minutes per day

2) How many days did your child have more than 10 minutes of moderate physical activity, which is equivalent in intensity to brisk walking or bicycling in the past week?

□ Not at all □ 1 day □ 2 days □ 3 days □ 4 days □ 5 days □ 6 days

□ 7 days (everyday)

| The examples of moderate physical activity: Volleyball, badminton, table tennis, swimming (slowly), doubles tennis, volume/fork dance, carrying light objects (except for walking) |
| --- |

How many minutes per day did your child usually do this moderate physical activity?

( ) hours ( ) minutes per day

3) How many days did your child go for a walk outside for at least 10 minutes at a time in the past week?

□ Not at all □ 1 day □ 2 days □ 3 days □ 4 days □ 5 days □ 6 days

□ 7 days (everyday)

While walking outdoors, how long does your child usually walk in a day?

( ) hours ( ) minutes per day

4) How many days did your child perform flexibility exercises such as stretching and free hand exercise in the past week?

□ Not at all □ 1 day □ 2 days □ 3 days □ 4 days □ 5 days □ 6 days

□ 7 days (everyday)

5) How many days did your child do muscle-strengthening activities like push-ups, sit-ups, dumbbells, weights, and iron bars in the past week?

□ Not at all □ 1 day □ 2 days □ 3 days □ 4 days □ 5 days □ 6 days

□ 7 days (everyday)

6) How many hours per day does your child watch TV on average?

Average of ( ) hours per day during weekdays,

Average of ( ) hours per day during the weekend

7) How many hours per day does your child spend time playing computers (including searching the Internet) or game consoles on average?

Average of ( ) hours per day during the weekdays,

Average of ( ) hours per day during the weekend

8) How many hours per day does your child sleep on average?

Average ( _) hours per day during the weekdays,

Average ( ) hours per day during the weekend
